# Free Fatty Acid Effects on the Atrial Myocardium: Membrane Ionic Currents Are Remodeled by the Disruption of T-Tubular Architecture

**DOI:** 10.1371/journal.pone.0133052

**Published:** 2015-08-14

**Authors:** Ryan P. O’Connell, Hassan Musa, Mario San Martin Gomez, Uma Mahesh Avula, Todd J. Herron, Jerome Kalifa, Justus M. B. Anumonwo

**Affiliations:** 1 Department of Internal Medicine, Center for Arrhythmia Research, University of Michigan Medical School, Ann Arbor, MI 48109, United States of America; 2 Department of Molecular and Integrative Physiology, University of Michigan Medical School, Ann Arbor, MI 48109, United States of America; University of Minnesota, UNITED STATES

## Abstract

**Background:**

Epicardial adiposity and plasma levels of free fatty acids (FFAs) are elevated in atrial fibrillation, heart failure and obesity, with potentially detrimental effects on myocardial function. As major components of epicardial fat, FFAs may be abnormally regulated, with a potential to detrimentally modulate electro-mechanical function. The cellular mechanisms underlying such effects of FFAs are unknown.

**Objective:**

To determine the mechanisms underlying electrophysiological effects of palmitic (PA), stearic (SA) and oleic (OA) FFAs on sheep atrial myocytes.

**Methods:**

We used electrophysiological techniques, numerical simulations, biochemistry and optical imaging to examine the effects of acutely (≤ 15 min), short-term (4–6 hour) or 24-hour application of individual FFAs (10 μM) on isolated ovine left atrial myocytes (LAMs).

**Results:**

Acute and short-term incubation in FFAs resulted in no differences in passive or active properties of isolated left atrial myocytes (LAMs). 24-hour application had differential effects depending on the FFA. PA did not affect cellular passive properties but shortened (p<0.05) action potential duration at 30% repolarization (APD_30_). APD_50_ and APD_80_ were unchanged. SA had no effect on resting membrane potential but reduced membrane capacitance by 15% (p<0.05), and abbreviated APD at all values measured (p≤0.001). OA did not significantly affect passive or active properties of LAMs. Measurement of the major voltage-gated ion channels in SA treated LAMs showed a ~60% reduction (p<0.01) of the L-type calcium current (I_Ca-L_) and ~30% reduction (p<0.05) in the transient outward potassium current (I_TO_). A human atrial cell model recapitulated SA effects on APD. Optical imaging showed that SA incubated for 24 hours altered t-tubular structure in isolated cells (p<0.0001).

**Conclusions:**

SA disrupts t-tubular architecture and remodels properties of membrane ionic currents in sheep atrial myocytes, with potential implications in arrhythmogenesis.

## Introduction

There is evidence that abnormal levels of plasma free fatty acids (FFAs) are associated with an increased risk of myocardial disease, including atrial fibrillation and heart failure.[1–3] A significant portion of the epicardial surface in large mammals is normally covered by adipose tissue, [4] and adipocytes may be involved in myocyte-adipocyte cross talk important in the physiological function of the myocardium [5]. One general postulate is that adipocyte derived biofactors (FFAs and adipokines, see Gong et al, 2003) are important signaling molecules, with the potential to remodel the myocardium when the biofactors are released in excess.[6] For instance, during and after myocardial ischemia there are increases in levels of FFAs in the coronary circulation.[7] Furthermore, there are increases in adipose deposits, extensive myocardial infiltration (*adiposis cardiaca*) and increased FA release during obesity. In the later case, the infiltration results in elevated levels of biofactors [5] (e.g. free fatty acids, interleukin-6, lipoprotein lipase, peroxisome proliferator-activated receptor-γ, among others), which by paracrine and “vasocrine” signaling pathways may overload the myocardium causing electrical and structural remodeling.[1,5,8]

The molecular and cellular mechanisms underlying myocardial remodeling are unknown. There is evidence, however, that FFAs cause abnormalities in the mechanisms of impulse initiation and propagation with inconsistent results from small animal models (rodent and lagomorph).[9–11] A recent study of an obese sheep model reported increased epicardial adiposity, abnormalities in atrial hemodynamics, and significant alterations in atrial impulse conduction, which contributed to an increased risk of AF.[12] However, the underlying cellular mechanisms were not determined. Biochemical composition of ovine and human epicardial fat are similar: the predominant fatty acids in epicardial fat depots are the saturated FFAs (SFAs; palmitic acid and stearic acid), and the mono-unsaturated fatty acid, oleic acid, with a composition respectively of 25%, 35% and 26%.[13] Stearic acid is the major saturated free fatty acid in ovine as well as human epicardial adipose tissue.[13–15,16] Although the electrophysiological effects of unsaturated fatty acids have been widely studied [17], SFA effects on myocyte electrophysiology are poorly understood. The objective of this study was to examine the effects, as well as the underlying mechanisms, of SFAs applied acutely on sheep atrial myocytes after isolation, or for 24 hours in cell culture.

## Materials and Methods

### Ethics Statement

All experiments were approved by the University Committee on the Use and Care of Animals at the University of Michigan (protocol 10552–2).

### Isolation of Adult Ovine Myocytes

Sheep left atrial myocytes were isolated using the Langendorff retrograde perfusion method as previously described [18–20] from normal young adult males (~30 kg). The protocol that was used conforms to the Guide for Care and Use of Laboratory Animals published by the United States National Institutes of Health (NIH) Publication No. 85-23, revised 1996. Briefly, male sheep (25–30 kg) were anesthetized with sodium pentobarbital (30 mg/kg I.V.). Following thoracotomy, the heart was immediately removed, transported from the necropsy room in ice-cold cardioplegic solution containing (in mmol/L): Glucose 280, KCl 13.44, NaHCO_3_ 12.6, Mannitol 34 similar to previously described.[18,21,22] The aorta was cannulated and retrogradely perfused (180 mL/min) with Tyrode’s solution containing (in mmol/L): NaCl 148, KCl 5.4, MgCl_2_ 1.0, CaCl_2_ 1.8, NaH_2_PO_4_ 0.4, Glucose 5.5, HEPES 15; pH 7.4 (NaOH) at 37°C until the effluent was clear of blood. Subsequently, a Ca^2+^ free solution containing (in mmol/L): NaCl 148, KCl 5.4, MgCl_2_ 1.0, NaH_2_PO_4_ 0.4, Glucose 5.5, HEPES 15; pH 7.4 (NaOH) was perfused for at least 10 minutes or until all contractions ceased. Collagenase (160 units/mL; Worthington Type II) was added to the Ca^2+^ free solution and perfused for 40 minutes. After cells were properly digested, a 1.5 cm x 1.5 cm region of the free wall bordering the posterior section of the left atrial chamber was collected for further dissociation. Sectioned tissue was then placed in Kraftbrühe (KB) solution [23,24] containing (in mmol/mL): KCl 80, MgSO_4_ 5, KH_2_PO_4_ 30, Glucose 20, EGTA 0.25, Creatine 5, β-Hydroxybutyric acid 5, Taurine 20, Pyruvic acid 5, ATP 5; pH 7.4 (KOH). Left atrial cells were isolated by gentle teasing and mechanical agitation. The isolated cells were kept at room temperature in KB solution for another 30 minutes before Ca^2+^ reintroduction [19]. Cells were centrifuged and re-suspended in a normal Tyrode solution at room temperature until use in acute (*t* = 0) experiments. In all other experiments, cells were centrifuged, plated and cultured in M199 medium with 3% penicillin/streptomycin, as previously described [25].

### Preparation of Free Fatty Acids Solutions

All solutions were prepared fresh for each experiment similar to previously described [26]. Stock solutions of 20% “fatty acid free” bovine serum albumin (Sigma) were dissolved in sterile DiH_2_O. We dissolved palmitic (Sigma), stearic (Sigma) or oleic acid (Sigma) in dimethyl sulfoxide (DMSO) to create a stock FA solution. Complexed FA:BSA solutions were generated to create a 6:1 molar ratio at 37°C. For example, 3 mM stearic acid was added to 0.5 mM BSA. This stock was added directly to either M199+ culture media (chronic experiments) or to normal Tyrode’s (acute experiments) to create a 10 μM working solution. Our vehicle control solution (referred to as CTL) was prepared similarly as our test conditions i.e., with BSA, DMSO and DiH_2_O, but lacked any FAs. The FA concentrations used in this study represent the total FA molar concentrations and are not the “calculated free” concentrations. The calculated free concentrations, using the methodology described in Richieri et al [27], was closer to ~25 nM for each FA. We chose these three FAs and the working concentration based on the findings in Haim et al [28], Richieri et al [27], Kim et al [16], Listenberger et al [29], Lin et al, 2014 [30] and Kang et al [31] among others that detail physiological concentrations in humans and sheep, along with differences in the application of FAs in cell culture vs. *in vivo* Importantly, our experimental conditions reflect FA concentrations that preclude lipotoxicity.[29]

### Single Cell Preparation for Experiments

Unless otherwise stated, experiments were conducted at 24 hours following cell isolation. Due to the high experimental variability in the outbred ovine model system, we have presented all experimental data with animal matched controls similar to Musa et al [18]. Furthermore, the analysis was conducted in this manner due to the inability to acquire all data sets within the experimental time frame.

### Single Cell Electrophysiology

The external solution, pipette filling solution, and protocols used for recording individual currents are detailed below.

### Current Clamp Experiments

Borosilicate glass electrodes were pulled using a Brown-Flaming puller (model P-97), yielding a tip resistance of 3–5 mΩ when filled with pipette solution. Action potentials were recorded on a 700B Multiclamp amplifier at 37°C (Molecular Probes) in normal HEPES Tyrode solution as previously described [20]. Briefly, cells were stimulated using a DS8000 Stimulator (World Precision Instruments) with 3–5 msec current pulses. Pulse trains of 20 stimuli were elicited at 1 Hz. Action potential duration values (APD30, 50, 80) were determined as the repolarization percent from the peak to baseline using custom software. All statistics (two tailed Student t tests) were carried out using Prism 6 (GraphPad).

External solution (mmol/L): NaCl 148, NaH_2_PO_4_ 0.4, MgCl_2_ 1, Glucose 5.5, KCl 5.4, CaCl_2_ 1.0, HEPES 15; pH 7.4 (NaOH).

Pipette filling solution (mmol/L): KCl 148, MgCl_2_ 1, EGTA 0, HEPES 5, Creatine 2, K_2_-ATP 5, Phosphocreatine 5; pH 7.2 (KOH).

### Voltage Clamp Experiments

Whole-cell recordings from isolated sheep atrial myocytes were done using standard methods [19]. All recordings were conducted at room temperature, and were performed using an Axopatch-200B Amplifier and/or 700B Multiclamp amplifier (Molecular Devices Sunnyvale, CA) and data acquisition and analysis were performed utilizing pClamp10.2 software (Molecular Devices Sunnyvale, CA). Pipette resistances ranged from 2–3 MΩ. Access resistance was compensated to 1–2 MΩ. Input resistance was 500 MΩ to 1 GΩ.

#### Sodium Current

External Solutions (mmol/L): NaCl 5, MgCl_2_ 1, CaCl_2_ 1.0, CdCl_2_ 0.1, HEPES 20, Glucose 11, CsCl 132.5 (pH = 7.35 with CsOH).

Pipette filling solution (mmol/L): NaCl 5, CsF 135, EGTA 10, MgATP 5, HEPES 5 (pH = 7.2 with CsOH).

Protocol: In order to record sodium currents cells were held at -160 mV followed by depolarizing steps from -80 to +10 mV in 5 mV increments. The duration of the voltage steps were 300 msec with a 5 second interval between successive voltage steps. Voltage-dependence of inactivation was assessed by holding at −160 to −40 mV followed by a 30-ms test pulse to −40 mV to elicit I_Na_.

Analysis: Voltage-dependent activation of I_Na_ was assessed by generating conductance voltage relationships (m-infinity curves) and fitting the data with a standard Boltzman function (Origin 8.1).

#### L-Type Calcium currents

External solutions (mmol/L): NaCl 137, CsCl 5.4, MgCl_2_ 1, CaCl_2_ 1.2, HEPES 10, Glucose 10, 4-Aminopyridine (4-AP) 2, (pH 7.35 with NaOH)[32].

Pipette filling solution (mmol/L): CsCl 120, TEA-Cl 20, MgCl_2_1, MgATP 5, Na_2_GTP 0.2, HEPES 10, and EGTA 10 (pH 7.2 with CsOH)[32].

Voltage dependence of peak I_Ca,L_ was measured by holding at -50mV; 300-msec voltage steps were applied from -40 to +60 mV in 5 mV increments. The interval between voltage steps was 3 sec. Voltage-dependence of inactivation was assessed by holding at -70 to +10 mV followed by a 30 msec test pulse to +10mV to elicit I_Ca_ and fitting the data with a standard Boltzman function (Origin 8.1).

#### Depolarization-activated potassium currents

External solutions (mmol/L): NaCl 148, NaH_2_PO_4_ 0.4, MgCl_2_ 1, Glucose 5.5, KCl 5.4, CaCl_2_ 1.0, HEPES 15 (pH 7.4 with NaOH). To block sodium channels and calcium channels 30 μmol/L TTX (Tetrodotoxin) and 5 μmol/L Nifedipine was added to the external solution.

Pipette filling solutuion (mmol/L): KCl 138, EGTA 10, HEPES 10, MgCl_2_ 1, glucose 5 (pH 7.4 with KOH).

Protocol: Potassium currents were recorded using 5-second depolarizing pulses to potentials between -40 mV and +60 mV from a holding of -70 mV. Voltage steps were in steps of 10 mV at 15 sec intervals [33].

### Antibodies

Primary antibodies used were: Rabbit polyclonal anti-Cav1.2 (Alomone Labs), Phospho Serine-1928 (Badrilla) and Rabbit polyclonal anti-S-nitrocysteine (Abcam), Mouse monoclonal sarcomeric α-actinin (Sigma). Secondary antibodies used were: Donkey anti-mouse Dylight 549, Donkey anti-rabbit Dylight 488 (Jackson ImmunoResearch), rabbit GAPDH antibody (Sigma-Aldrich).

### Immunofluorescence

Immunofluorescence analysis was carried out on myocytes plated on 22 mm glass coverslips. Cells were fixed with 3% paraformaldehyde in PBS, and then blocked with 10% Normal Donkey Serum in 0.1% Triton-X100 in PBS (NDS) for 1 h at room temperature. Incubation with primary antibodies was done in 5% NDS, overnight at 4°C. The next day coverslips were washed with 0.1% Triton-X100 in PBS (PBS-T, 3 x 10 min) and incubated for 90 minutes at room temperature with secondary antibodies diluted in 5% NDS. Coverslips were then washed with PBS-T (3 x 10 min) and PBS (1 x 10 min). Samples were treated with with 4',6-Diamidino-2-Phenylindole, Dihydrochloride (DAPI), a nucleus marker, (Molecular Probes) and then mounted onto slides using FluoromountG (SouthernBiothech). Immunostained preparations were analyzed by confocal microscopy (Nikon A1R) to determine protein localization in relation to cell morphology. Line scanning analysis of 20 μm sections was conducted similar to Musa et al [18].

### SDS-PAGE and Immunoblotting

Control and treated cardiac myocytes were washed in cold PBS, lysed directly in the modified loading buffer (Tris•HCl, 25 mmol/l; NaCl, 150 mmol/l; EDTA, 1 mmol/l; NaF, 4 mmol/l; Sodium ortho-vanadate, 2 mmol/l; 1% Triton X-100, protease inhibitor, 5% glycerol, 1%SDS, 0.05%bromophenol blue, 5% β mercaptoethanol) and sonicated. Lysates (20 μL) were separated by gel electrophoresis in one-dimensional 4–20% sodium dodecyl sulfate polyacrylamide gel (Invitrogen) in Tris-Glycine buffer (Fisher). Separated proteins were transferred to nitrocellulose (Bio-Rad, 0.45 μm pore size) in a Hoeffer transfer apparatus. Nonspecific binding sites were blocked by incubation with 5% nonfat dry milk (NFM) in PBS with 0.05% Tween-20 (PBS-T). Membranes were then incubated with specific primary antibodies (0.5–1 ug/ml) diluted in 5% NFM overnight at 4°C. Following four ~5 minute washes in PBS-T, membranes were incubated with horseradish peroxidase (HRP)-conjugated secondary antibodies (Jackson Immunorersearch) for roughly one hour. Antigen complexes were visualized using enhanced chemiluminescence (Pierce). Protein bands (top bands, were doublets are present) were quantified by digital densitometry with a BioRad Fluor-S imager and Quantity One software (Bio-Rad). Precision Plus Protein All Blue Standards (Bio-Rad) was used to determine molecular weight of all blots. GAPDH was used as a control in all experiments.

### Transverse Tubule Imaging

Protocol: 1 mg of voltage-sensitive dye Di-8-anepps (Life Technologies, Carlsbad, CA) was dissolved in 1 mL of DMSO and used as a stock (stored at 4°C). Working solution was prepared as a 50:50 v/v mix of di-8-anepps stock and 20% pluronic acid as previously described [34]. 15 μL of stock solution was used per 1 mL 300 μM Ca^2+^ Tyrode solution. Myocytes were allowed to incubate for 10 min before imaging.

Imaging: Imaging of t-tubules was done on a confocal microscope (Nikon A1R) using a 60x oil immersion objective. Three-dimensional rendering of atrial and ventricular myocytes was constructed by stacking 15–25 XY planar images from the superior to the inferior lateral membrane. Images were acquired with 0.5 μm spacing. Confocal images were visualized and analyzed with the NIS-Elements program (Nikon).

Analysis: To determine the extent of the t-tubular network within a given cell we analyzed a single z-slice at the midline of the cellular z-axis. We manually created a region of interest (R1) inside the periphery of the cell (excluding the lateral membrane and intercalated disk). A second region of interest was created just outside the border of the lateral membrane and the intercalated disk and encapsulated the entire cell (R2). These regions were quantified by pixel intensity summation in NIS-Elements (Nikon, Japan). A third region of interest was created in the nuclear region (R3). The area of R3 was extrapolated to the surface areas of R1 and R2 and was used to correct the data for background fluorescence. After correction, we took the ratio of R1/R2 as a measure of t-tubular content in a given cell. All 2-D imaging and analysis was done on the core of the myocyte after establishing vertical sarcolemmal membranes for Z-stack imaging. This analysis was repeated individually for each cell and a mean ratio ± SEM was calculated for each group.

External solution (mmol/L): NaCl 148, NaH_2_PO_4_ 0.4, MgCl_2_ 1, Glucose 5.5, KCl 5.4, CaCl_2_ 0.3, HEPES 15; pH 7.4 (NaOH).

### Statistical Analyses

Data are presented as the mean ± SEM. Ca_V_1.2 and alpha actinin periodicity was determined by linear regression fits o f the data. APD analyses, voltage clamp experiments and calcium transient data and alpha smooth muscle actin western data used a two tailed unpaired Student t-tests and were carried out using Prism 5 (GraphPad) or Excel (Microsoft). Significance was defined by p-values ≤0.05.

## Results

### Fatty acid effects on atrial myocyte electrophysiology and structure

#### Fatty acid effects on resting membrane and action potential properties

**Fig 1:**
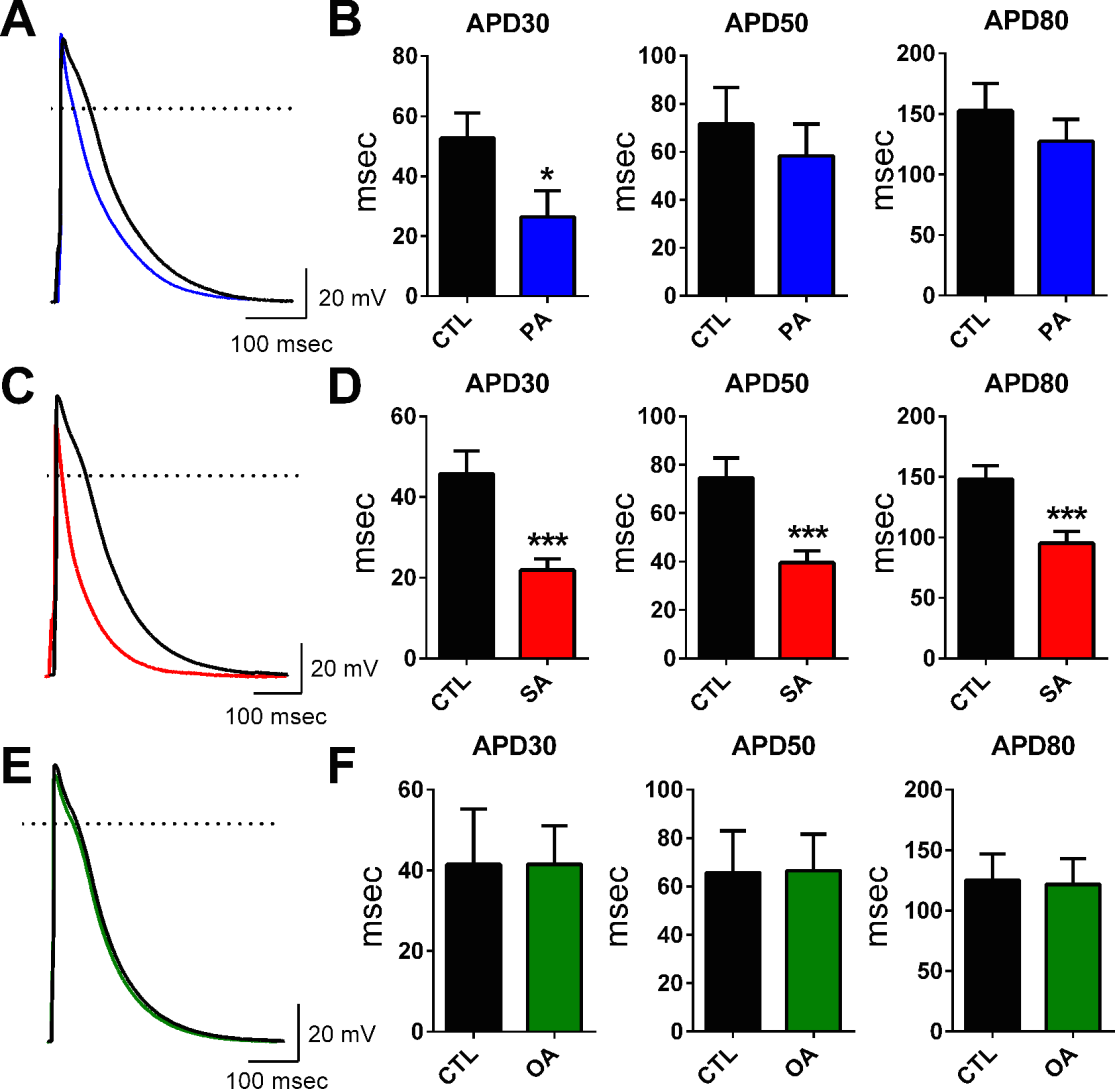
Saturated free fatty acids, but not a mono-unsaturated fatty acid, shorten action potential duration in atrial cells. Panels A, C, E; Atrial myocyte action potential recordings in control solution (CTL, black) and following incubation in solutions containing 10 μm palmitic acid (PA, blue), stearic acid (SA, red) or oleic acid (OA, green). Average APD30, 50, & 80 measurements in CTL and following incubation in PA (Panel B, n = 9, 12; *p<0.05), SA (Panel D, n = 22, 24; ***p<0.001) and OA (Panel F, n = 6, 5). CTL solution in this and subsequent Figs, represent vehicle control, see Methods section. Scale bars: 100 msec and 20 mV.

In all electrophysiology experiments, myocytes were isolated from the left atrial (LA) appendage region of adult sheep hearts (areas depicted in S1 Fig). We studied the effects of 24-hour incubation of left atrial myocytes (LAMs) in SFAs (PA, SA) or in OA at 10 μM (see Methods). PA and OA did not alter passive electrical properties (RMP, cell cap, etc.; Table 1). SA, similarly, had no effect on RMP but significantly reduced whole cell capacitance (p<0.05). The values presented in Table 1 (i.e. after 24 hrs) were not different from the passive properties collected acutely (0 hrs; data not shown). Fatty acid effects on APD were examined at three values of repolarization (30%, 50% and 80%) and illustrated in Fig 1. PA significantly reduced APD30 (msec) from 52.7 ± 8.4 to 26.4 ± 8.8; p<0.05 (Fig 1A and 1B). Incubation of myocytes in SA resulted in a very significant reduction (p≤0.001) of APD at all values measured (Fig 1C and 1D). In contrast to the effects of the SFAs, the mono-unsaturated OA did not alter APD properties at any value of repolarization measured (Fig 1E and 1F). Initially, we tested the effects of acute FA exposure similar to previous studies [28,30,31] and found no changes to LAM APD. Results for PA and SA are shown in S2 Fig. Panels A-D. During our acute experiments we increased our FA concentration to 50 μM and did not observe any remodeling in current or voltage clamp experiments (data not shown). We also measured short-term (4–6 hr) incubation and did not observe any alterations in myocyte APD. Data for SA is presented in S2 Fig Panels E-G.

**Table 1:** Electrophysiological properties of atrial myocytes incubated in palmitic (PA), stearic (SA), oleic (OA) acids and vehicle control (CTL).

	RMP (mV)	AP Amplitude (mV)	AP Overshoot (mV)	dv/dt_max_ (mV/msec)	Cell Cap (pF)
**CTL n = 9; N = 3**	**-78.7 ± 0.90**	**106.5 ± 3.63**	**28.0 ± 3.44**	**227.9 ± 12.6**	**59.2 ± 2.94**
**PA n = 12; N = 3**	**-79.5 ± 0.81**	**118.8 ± 4.20**	**36.3 ± 3.26**	**231.8 ± 19.4**	**63.7 ± 5.15**
**CTL n = 22; N = 5**	**-79.2 ± 0.6**	**126.6 ± 2.52**	**34.0 ± 2.04**	**223.9 ± 13.1**	**63.1 ± 3.08**
**SA n = 24; N = 5**	**-80.3 ± 0.7**	**119.0 ± 2.92**	**34.3 ± 2.54**	**210.0 ± 13.8**	53.8 ± 2.55*
**CTL n = 6; N = 2**	**-77.8 ± 0.74**	**119.0 ± 2.68**	**37.3 ± 1.92**	**192.5 ± 18.3**	**71.1 ± 5.98**
**OA n = 5; N = 2CTL n = 6; N = 2**	**-79.5 ± 1.21–77.8 ± 0.74**	**123.2 ± 4.02119.0 ± 2.68**	**38.0 ± 2.9237.3 ± 1.92**	**199.7 ± 12.0192.5 ± 18.3**	**64.9 ± 2.7971.1 ± 5.98**
**OA n = 5; N = 2**	**-79.5 ± 1.21**	**123.2 ± 4.02**	**38.0 ± 2.92**	**199.7 ± 12.0**	**64.9 ± 2.79**

Acquisition of Resting Membrane Potential (RMP), Action Potential (AP) Amplitude, Action Potential Overshoot, dv/dt_max_, and Cell Capacitance by patch clamp is presented with animal matched controls (CTL). (*p<0.05).

### Fatty acid effects on membrane ionic currents

**Fig 2:**
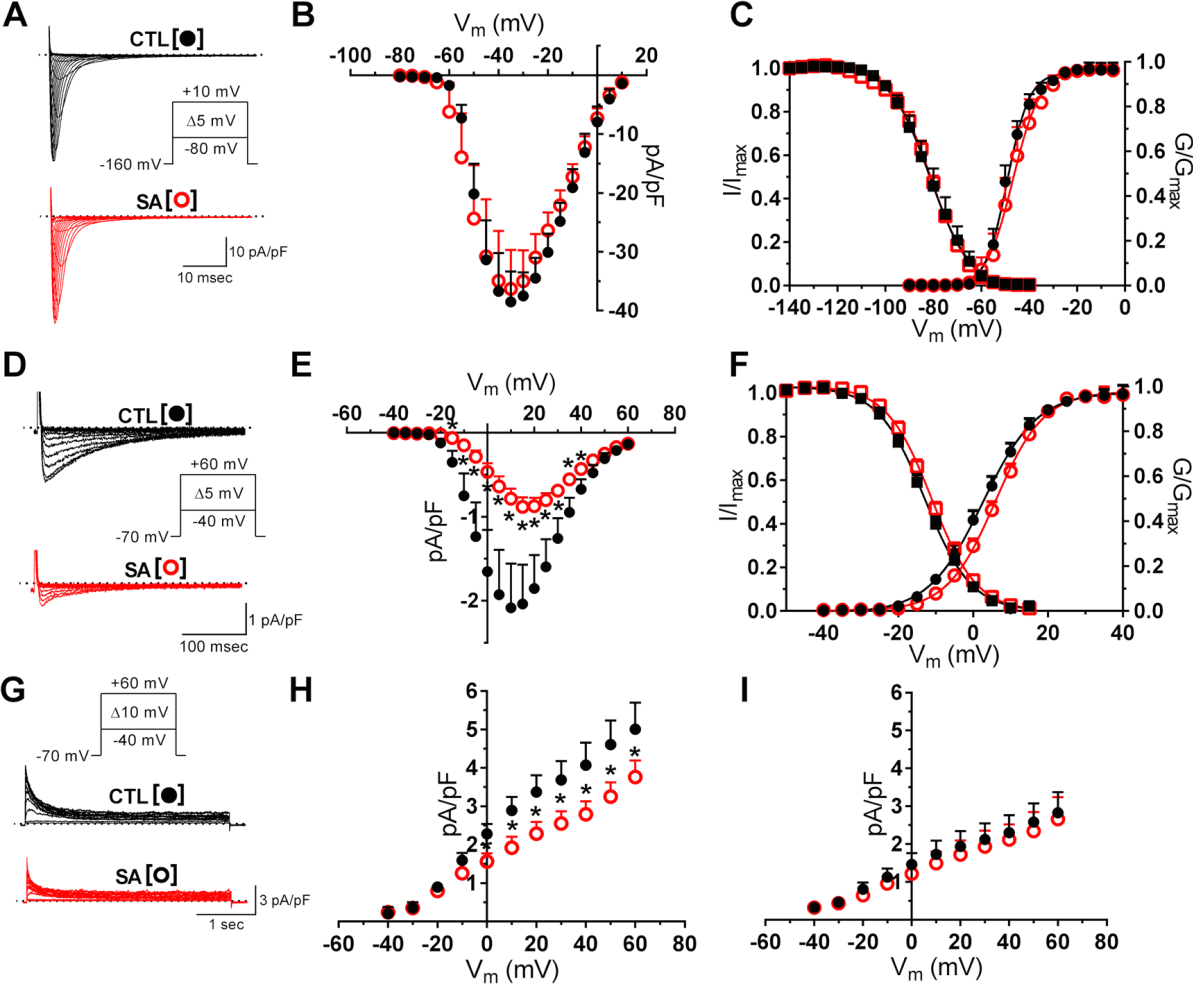
Voltage-gated ionic currents are differentially affected by stearic acid. Panel A: Representative traces of sodium currents (I_Na_) in control (CTL, black) and in SA (10 μm; red) treated cells. Inset: voltage-clamp protocol for current activation. Plots of current density (Panel B), and of voltage-dependence of current activation and inactivation (Panel C), n = 5, 7). Panel D: Representative traces (selected) of calcium current (I_Ca, L_) in CTL and in SA (10 μm; red) treated cells. Inset: voltage-clamp protocol for current activation. Plots of current density (Panel E), and of voltage-dependence of current activation and inactivation (Panel F). Incubation of myocytes in SA caused a significant reduction of I_Ca, L_ density (n = 24, 22; *p<0.05). Panel G: Transient outward potassium currents (I_TO_) in CTL and in SA treated cells. Inset: voltage-clamp protocol. Panel H: Current-density measurements showing effects of SA on peak I_TO_, but not on the steady state current (I_SS_) (Panel I) (n = 8, 8; *p<0.05).

Results from the preceding section show that compared to PA, SA caused more significant changes on the passive and active electrical properties of LAMs. An examination of free fatty acid effects on major membrane currents was therefore carried out in cells incubated primarily in SA. In these experiments, we examined the effects of 24 hour application of SA on the fast sodium current (I_Na_) and the L-type calcium current (I_Ca-L_), which are the key voltage-gated currents involved in membrane depolarization in atrial cells. SA did not alter I_Na_ current density (Fig 2A and 2B), or the voltage dependent activation or inactivation properties (Fig 2C). In contrast, SA decreased peak I_Ca-L_ by ~60% compared to control values (Fig 2D and 2E). Peak I_Ca-L_ density was reduced from -2.2 ± 0.5 pA/pF to -0.9 ± 0.1 pA/pF (p<0.01). As shown in Fig 2F, the reduction of I_Ca-L_ occurred without changes in voltage-dependent current activation or inactivation properties. We also examined free fatty acid effects on the voltage-gated, transient outward current (I_TO_), which is major repolarizing potassium current in ovine atrial cells.[35] The analysis of SA effects focused on peak I_TO_ and the steady-state current (I_SS_). Compared to control, SA significantly reduced I_TO_ density, which was measured at voltages in the range of -40 mV to +60 mV (Fig 2G and 2H). At +40 mV for example, there was a ~30% reduction of current density from 4.1 ± 0.6 pA/pF to 2.6 ± 0.3 pA/pF (p<0.05). However, the SA incubation did not change I_SS_ density (Fig 2I).

### Remodeled atrial action potential simulations

**Fig 3:**
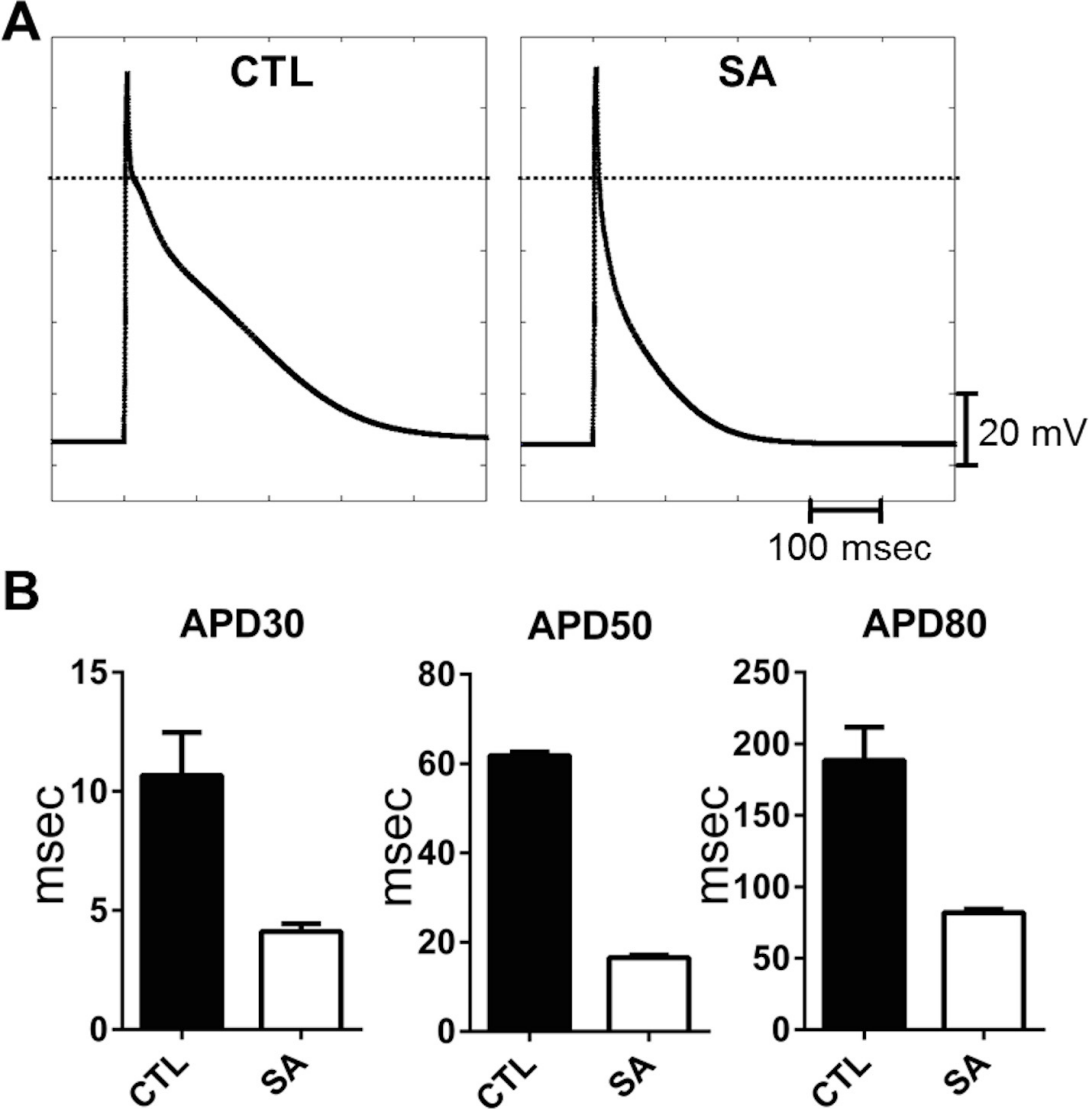
Human atrial action potential model simulation of stearic acid effects on ionic currents in isolated atrial cells. Panel A: Model generated action potential under control conditions (left) and following SA-induced changes in ionic currents (see text for details) (right). Panel B: Measurements of APD30, 50 & 80 from ten consecutive action potential simulations under control (CTL, black) and with SA treatment (white).

Our results on the SA induced inhibition of I_Ca-L_ and I_TO_ are expected to differentially modulate atrial cell action potential, by an abbreviation (I_Ca-L_), or a prolongation (I_TO_), of APD. Although the current density measurements (Fig 2) would suggest a predominant effect of the former, it was not possible in the experiments to determine whether the SA effects on these ionic mechanisms were sufficient to explain the abbreviation, or whether other (ionic) mechanisms were necessary. Numerical simulations were therefore used to further investigate these possibilities. We incorporated the experimental results (of Fig 2) into a previously published human atrial cell model.[36] Currents in the presence of SA were introduced in the simulations by decreasing peak I_Ca-L_ (by 60%) and I_TO_ (by 30%) compared to their respective control values. The model-generated action potentials, under control and with SA treatment, are shown respectively in the left and right panels of Fig 3. Results from the simulations showed that these changes in ionic current densities were sufficient to reproduce the reduced APD values (APD30, APD50 and APD80) measured experimentally. For example, control APD80 was reduced from 188.6 ± 7.7 msec to 82 ± 0.7 msec. Our simulation results are thus consistent with the results of our cell electrophysiology experiments in isolated LAMs. Additional simulations were conducted with reductions in I_Ca-L_ or I_TO_ individually, which confirmed I_Ca-L_ as the major current responsible for shortening atrial APD (data not shown). These findings are similar to the observations reported by Martins et al [22], which investigated experimentally and with numerical simulations, the electrophysiological alterations of an ovine model of persistent atrial fibrillation where both I_Ca-L_ and I_TO_ are reduced significantly, resulting in a shortened APD.

### Stearic acid effects on Cav1.2 protein

**Fig 4:**
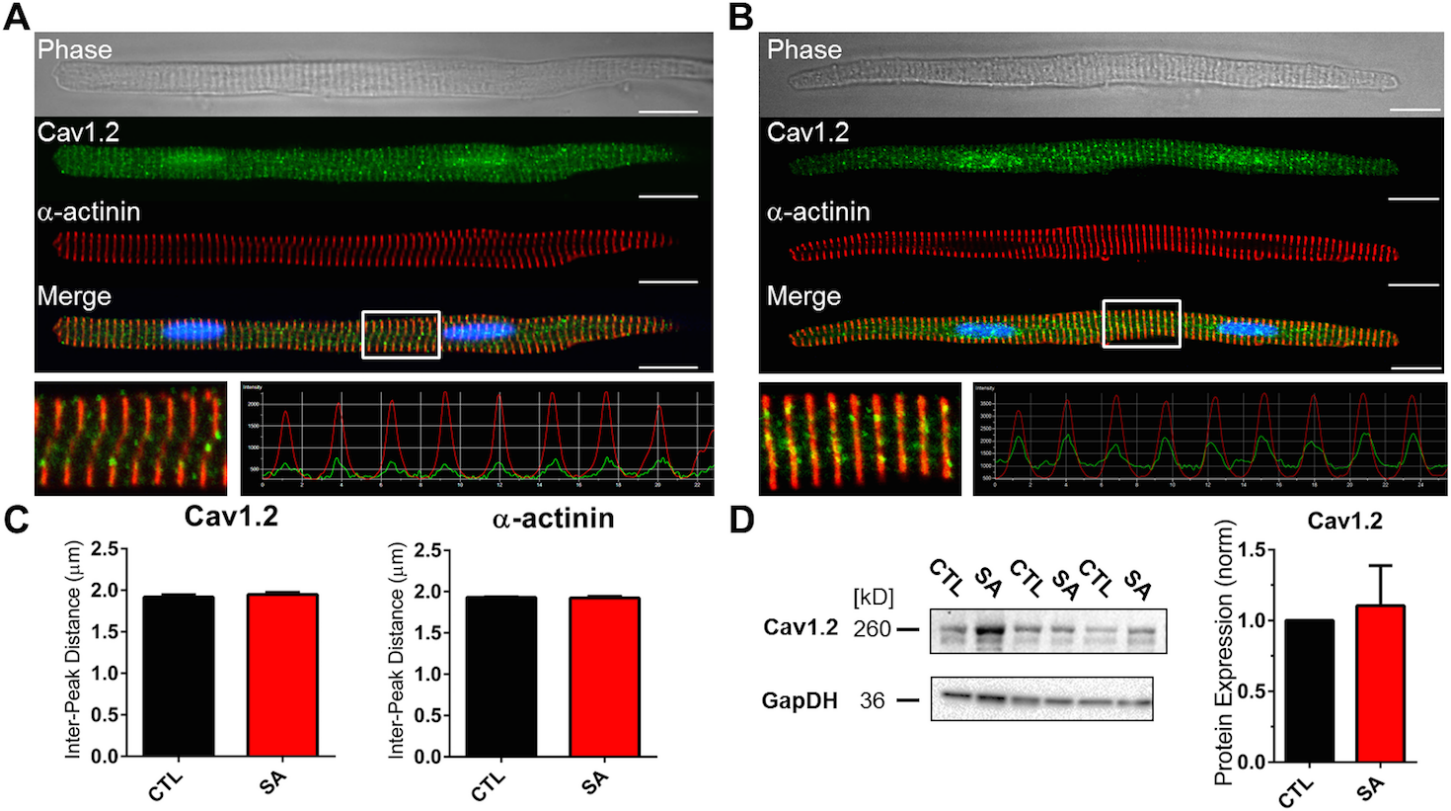
Cav1.2 Localization and whole cell protein content is not affected by stearic acid. Transmitted light image of an atrial myocyte (top), Cav1.2 protein staining (green), α-actinin staining (red) and a merged image of Cav1.2, α-actinin and DAPI staining (bottom) under control (CTL; Panel A) and following incubation in stearic acid (SA; Panel B). Inset: 20 μm section of the merged image and corresponding intensity profile of Cav1.2 and α-actinin. Scale bars 20 μm. Panel C: Quantification of the intensity profiles shows SA did not alter the mean distance between intensity peaks for Cav1.2 (left) or α-actinin (right; n = 12, 12). Panel D: (left) Western blot for Cav1.2, with GAPDH as a control. Pane D: (right) normalized densitometry plot of Cav1.2 protein levels in CTL and SA treated cell lysates (n = 3).

Our investigations on LAM electrophysiology show that the major effect of SA was on I_Ca-L_ density. We therefore used a combination of biochemical and optical techniques to examine SA-induced changes on Cav1.2 protein, the molecular correlate of the underlying channel. Labeling of Cav1.2 and α-actinin Fig 4A–4C shows that SA did not alter Cav1.2 protein localization, i.e., relative to the sarcomeric α-actinin pattern, and was distribution of the Cav1.2 protein was similar to what has been previously described.[37,38] Cav1.2 remains in close proximity to the Z-lines, and the spacing of the Cav1.2 intensity peaks (and of α-actinin) were similar and unchanged by SA treatment (Fig 4C; n = 12, 12). Furthermore, atrial cell Cav1.2 protein levels were not significantly affected by incubation in SA (Fig 4D; N = 3). These data suggest that alterations in cellular Cav1.2 protein levels is not the mechanism driving the SA-induced changes in I_Ca-L_. Channel nitrosylation or phosphorylation are well described post-translational mechanisms for I_Ca-L_ regulation.[39,40] In additional sets of experiments (see Online Supplement), we investigated the potential roles of these mechanisms by immunocytochemistry and western blot shown in S3 and S4 Figs. Results from these experiments showed no differences between nitrosylation or phosphorylation of Cav1.2 in control and SA treated cells.

### Fatty acid effects on t-tubular structure

**Fig 5:**
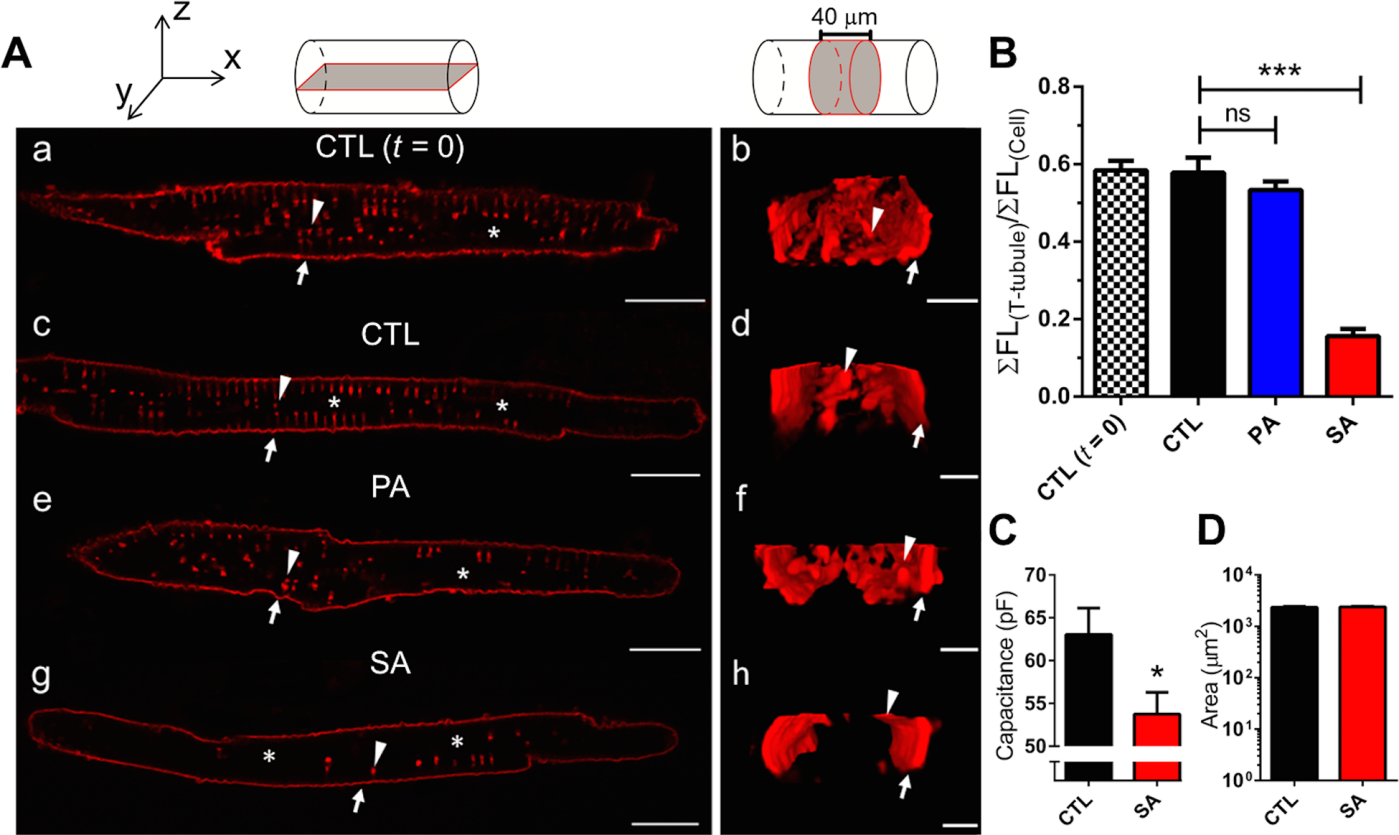
Stearic Acid disrupts t-tubules in atrial myocytes. Panel A (top): Coordinate axes for reference and schematic diagrams illustrating the fields of view. Panel A: subpanels (a, c, e & g) are XY planar views and (b, d, f & h) are 40 μm ZY cross-sectional views of the same cell. (a & b) Di-8-ANEPPS staining of t-tubules in freshly dissociated (*t* = 0) atrial myocytes (n = 19). After 24 hrs in culture, Control (CTL) cells shown in (c) and (d) retain t-tubule structures (n = 20). (e) and (f) T-tubular structure after chronic incubation of myocytes in PA (n = 19). (g) and (h) reduction in t-tubules after 24 hr incubation of myocytes in SA (n = 25). Arrows indicate the lateral membrane and arrowheads highlight an individual t-tubule in both views. (*) identifies the nuclear region. Scale bars: 20 μm (XY) and 5 μm (ZY). Panel B: Quantification of t-tubules in using the ratio of the t-tubule region and total cell fluorescence. SA reduced the presence of t-tubule structures in LA myocytes (CTL vs. SA; ***p<0.0001, n = 25), CTL vs. PA; p = ns (n = 19)). Panel C: SA reduced the capacitance of atrial myocytes following incubation in SA (Panel C, n = 83 *p<0.05), but did not alter 2D surface area of myocytes (n = 68).

The membranes of transverse (t)-tubules are well-recognized microdomains for the cellular localization of Cav1.2, and their disruptions have been reported to result in a reduction of I_Ca-L._[41,42] We hypothesized that free fatty acid induced changes in t-tubular architecture or structural integrity could provide an explanation for the reduction in I_Ca-L_ given the lack of alterations to Cav1.2 protein localization and abundance. To examine this possibility, LAMs in control or incubated with PA or SA for 24 hours, stained with Di-8-ANEPPS and visualized by confocal microscopy. In Fig 5A, the insets at the top illustrate the area of view for the left (a, c, e, & g) and right (b, d, f, & h) panels. The left panel is an XY single image and the right is a 40 μm ZY cross-sectional area viewed from the longitudinal end of the cell. Initial control experiments showed no difference between t-tubule structure in atrial cells acutely after cell isolation (*t* = 0; panels a & b) and 24 hours in control media (CTL; panels c & d). The imaging results show that cells incubated in PA had a slight observable reduction of t-tubular staining (Fig 5Ae and 5Af). In contrast, cells incubated with SA demonstrated a dramatic reduction in the visualized t-tubule network (Fig 5Ag and 5Ah). T-tubule pixel intensity at the midline of the cellular z-axis was quantified (excluding lateral and intercalated disc membranes), summed and normalized to total cellular intensity (see Methods) and plotted in Fig 5B. Compared to control cells, SA caused a ~4-fold decrease in the intensity of normalized t-tubular staining (0.58 ± 0.04 versus 0.16 ± 0.02; ***p<0.0001). It is generally acknowledged that t-tubule membranes account for 15–50% of the capacitance of cardiac ventricular myocytes.[34] Consistent with this reduction of t-tubular staining in SA treated atrial cells, we observed a >15% decrease in whole cell capacitance (Table 1, Fig 5C). In addition, we analyzed the basic morphometry of myocytes and found the 2D cell surface area to be no different between control and cells incubated with SA (Fig 5D). These data show that the reduction in stained t-tubular membranes is a possible mechanism for the reduction of I_Ca-L_ observed with incubation of cells in SA. Additional control experiments (S5 Fig) established our imaging technique resolved the previously reported differences between atrial and ventricular t-tubular architectures in large mammals.[34]

## Discussion

In humans and in large mammals, epicardial fat distribution on the atrial myocardium is considered generally less extensive compared to the distribution on the ventricular epicardium. In the left atrium of sheep and human hearts, however, the pulmonary vein region is reported to have extensive fat deposits and has been suggested to contribute to arrhythmogenicity (S1 Fig).[11,43] There have been notable studies addressing the acute modulation of ventricular myocyte electrophysiology in cell lines and in rodents, but this study is the first to address FA induced remodeling in the atria of a large animal model. Our choice of the ovine model is predicated on the similarity to humans, of the biochemical content of epicardial fat as well as the ionic currents underlying myocardial excitation. We investigated the changes in electrical and structural properties of ovine atrial myocytes following incubation of the cells in each of the three major FAs found in the ovine (PA, SA, OA)[13]. Whereas the monounsaturated OA had no measurable effects, the fully saturated PA and SA caused significant changes in myocyte electrophysiological and structural properties, with SA also resulting in structural alterations.

### Remodeling of atrial myocyte electrophysiology

In a previous study [28], the effects of palmitate on murine ventricular myocyte excitability properties were investigated under acute exposure of the fatty acid.[28] The major findings from this study was that palmitate shortened APD, an abbreviation that was the result of an ~20% increase in the voltage-gated potassium currents, consisting of I_to,f_, I_K,slow_ and I_ss_.[28] In the ovine model, acute or short-term (4–6 hr) exposure to SFAs had no effects on LAMs (S2 Fig). We then tested the major FAs in ovine epicardial adipose tissue individually at 24 hours. It is indeed possible that longer incubations with FFAs would cause either structural and/or electrophysiological changes not seen in a 24 hour time period. However we attempted to minimize both changes due to remodeling in culture as well as maintain the viability of our experimental myocytes, which begin to deteriorate after >24 hours in culture (48 hour data not shown). In an ideal sense these experiments would be done in obese animals allaying many of these weaknesses but this model was not available for this study.

The FA effects we report here, primarily an ~60% reduction of I_Ca-L_ by SA, are different from those previously described [28], presumably reflecting species dependent differences (murine vs. ovine), regional differences (ventricular vs. atrial), and the nature of the FA challenge (acute vs. chronic) compared to studies involving PA & OA [28,44]. Our simulation showed that these changes in ionic currents reproduced changes in APD measured experimentally. We chose to focus on I_Ca-L_ rather than I_TO_ since the predominant electrophysiological effect of SA was shortening of AP duration. We would expect that a reduction in I_TO_ to lengthen, rather than shorten, the AP duration. Therefore, we concluded that the effect of reduced I_Ca-L_ was more significant in altering atrial cell electrophysiology. Furthermore, the CaV1.2 protein is known to localize to the T-tubules [45], which we show in our experiments are significantly disrupted by FFA treatment. This observation is thus consistent with a significant role of FFA effects on I_Ca-L_ in reducing atrial AP duration. Additionally, simulations performed on a model of persistent atrial fibrillation [22] observed similar reductions in I_Ca-L_ and I_TO_ at the cellular level and demonstrated that the individual contribution of reduced I_Ca-L_ alters APD morphology to a significantly greater degree than a reduction in I_TO_ (see Martins et al, 2014; Online Supplement).

We surmise OA, the only unsaturated FA tested, did not alter LAM electrophysiology due to the structural differences between fully saturated versus unsaturated FAs. The inability of OA to alter current density of ion channels has been shown previously [44]. More interestingly are the differences in alterations by PA and SA. While we did observe a reduction in APD30 via PA incubation, we did not observe a significant change in t-tubular structure. Given PA’s involvement in subcellular trafficking (e.g. palmitoylation) it is likely the PA induced changes utilize an entirely separate mechanism of remodeling. This is further supported by the differences in the cellular metabolism of OA & PA vs. SA, which may contribute to the differences in electro-structural remodeling observed in this study [46]. However, we did not test this hypothesis directly.

### Remodeling of t-tubular architecture

It is generally accepted that the atrial t-tubule network is fairly prominent in large mammals, and has profound effects on spatio-temporal properties of systolic Ca^2 +^ transients.[47] Importantly, t-tubules have been implicated in a number of pathological conditions affecting the heart, and given the predominant localization of Cav1.2 channels in the t-tubule network [39], it was recently suggested that reduced coupling between influx of Ca^2+^ and the release of sarcoplasmic reticulum (SR) Ca^2+^ play a role in the arrhythmogenesis in an ovine AF model.[41] Furthermore, it was shown in swine that t-tubule heterogeneity exists across the left and right atria and the density of I_Ca-L_ corresponds directly to the presence of t-tubules.[48]

Overall, biochemical analysis in this study shows that with SA incubation, Cav1.2 proteins remain localized, with similar total protein content, to t-tubular spaces within the interior of the cell and showed no apparent changes in α-actinin periodicity. In our experiments, SA caused a significant reduction of the visible atrial t-tubule network and cell capacitance, without a change in cell morphometry. Taken together, these data suggest SA induced modification does not disrupt the overall t-tubular network but likely causes a modification near the lateral membrane at the t-tubule invaginations (Z-groove) similar to the observations made in Lyon et al [49]. By ‘pinching off’ or physically occluding the t-tubule lateral membrane pore, SA incubation thereby prevents access of Di-8-ANEPPS to the network of intact tubules located within the core of the myocyte. Thus, Cav1.2 proteins remain localized to intact t-tubules in the core but are not activated during stimulation (excitation-contraction coupling) and therefore compromise calcium-handling mechanisms via a reduction in I_Ca-L_. It has been suggested that during metabolic stress, t-tubule remodeling may be the consequence of mitochondrial enlargement, which causes the occlusion of the t-tubular lumen [50]. In cardiac myocytes, mitochondria are the site of FA oxidation and diets causing elevated serum FA levels lead to mitochondrial stress and enlargement (MtSE)[51,52]. It has been demonstrated previously that stearic and palmitic acid have differential metabolic pathways in large mammals and may underlie the differences in each treatment on t-tubule remodeling demonstrated here compared to studies in the murine model [46].

## Limitations

In the present study, we have focused our experiments in evaluating one group of biofactors, the FFAs present in the epicardial adipose tissue and serum of the ovine atrial myocardium. The major limitation of this study is the *in vitro* nature of our experiments. *In vivo*, biofactors produced by adipocytes therein may regulate the atrial myocardium by mechanisms involving additive effects of these (and other) fatty acids. It has been shown that specific combinations of fatty acids found in blood serum under normal conditions can have physiologically beneficial effects to cardiac structure and function [53]. The combinatory effects of these fatty acids do warrant investigation but are beyond the scope of the present study. Also, it is expected that the response of cardiac myocytes to the incubation in the fatty acids may be different if the contractile machinery were fully activated during cardiac muscle contraction.

## Conclusions

24-hour incubation of saturated FFAs leads to the remodeling of the normal architecture of t-tubules and the properties of membrane ionic currents in ovine atrial cells. 24-hour application of stearic acid modifies the t-tubular network, primarily affecting functional properties of I_Ca-L_, with potential implications in electrical excitation of the atrial myocardium.

## Supporting Information

S1 Fig Epicardial fat tissue distribution on the ovine heart. Panel A: Normal adult sheep heart (Langendorff retrograde perfusion), showing epicardial (atrial and ventricular) fat deposits. (LAA; left atrial appendage LV; left ventricle EF; epicardial fat on atrial and ventricular surfaces). Green scale (cm) is shown close to the posterior wall, adjacent to the openings of the pulmonary veins. Yellow box is 2 x 3 cm. Panel A1: (inset from Panel A) oval and rounded rectangles represent regions of dense atrial epicardial fat. Panel B: LAA tissue section: Note significant epicardial fat layer with extensive adipocyte infiltration of the left atrium. Scale bar: 1mm. Panel B1: Tissue section from yellow inset in panel B showing myocytes (MYCTS), isles of adipocytes, and collagen (CLG) stained with Picoserius. Scale bar: 50 μm.
(TIFF)

S2 Fig Acute exposure to free fatty acids has no effect on atrial myocytes. Panel A: Representative action potential recordings under acute fatty acid application of control (CTL; black) and of PA (blue) solutions. Panel B: Quantification of APD30, 50 & 80 values (CTL: n = 13, PA: n = 15). Panel C: Representative action potential recordings under acute fatty acid application of control (CTL; black) and of SA (red) solutions. Panel D: Quantification of APD30, 50 & 80 values in CTL and SA treated cells (n = 13, 13). Panel E: Representative traces of cells incubated 4–6 hrs with CTL (black) and SA (red). Quantification of APD30, 50, 80 values showed no difference between both groups (n = 7, 9). Scale bars: 100 msec and 20 mV.
(TIF)

S3 Fig Cav1.2 nitrosylation is not affected by stearic acid. Panel A/B: CTL and SA immunofluorescence images of transmitted light, Cav1.2 nitrosylation (Cav1.2N), α-actinin and a merged image (n = 23, 20) Scale bars: 20 μm. Panel C: Quantification of the intensity profiles shows SA did not alter the mean distance between intensity peaks for Cav1.2N (left) or α-actinin (right; n = 12, 12). Mean distance between Cav1.2N and α-actinin peaks were similar in both groups and unchanged from CTL to SA. Panel D: (left) Western blotting for Cav1.2N with GAPDH as a control. Pane D: (right) normalized densitometry plot of Cav1.2N protein levels in CTL and SA treated cell lysates (N = 3).
(TIF)

S4 Fig Cav1.2 phosphorylation at serine 1928 is not affected by stearic acid. Panel A/B: Immunofluorescence images of transmitted light, Cav1.2 phosphorylation (Cav1.2P), α-actinin and a merged image under control (CTL) and stearic acid (SA) treatment (n = 24, 22). Scale bars: 20 μm. Panel C: Quantification of the intensity profiles shows SA did not alter the mean distance between intensity peaks for Cav1.2P (left) or α-actinin (right; n = 12, 12). Mean distance between Cav1.2P and α-actinin peaks were similar in both groups and unchanged from CTL to SA. Panel D: (left) Western blot for Cav1.2P with GAPDH as a control. Pane D: (right) normalized densitometry plot of Cav1.2P protein levels in CTL and SA treated cell lysates (N = 3).
(TIF)

S5 Fig Comparison of T-tubule structure in sheep atrial and ventricular myocytes. Panel A (top): Coordinate axis for reference and schematic diagrams illustrating the fields of view. Panel A: subpanels (a) and (c) are XY planar views and (b) and (d) are 40 μm ZY cross-sectional views of the same cell. (a) and (b) Di-8-anneps stain of T-tubules in a freshly dissociated (*t* = 0) left ventricular myocyte (LVM) which contains an extensive T-tubule network. For comparisons, (c) and (d) show a freshly dissociated (*t* = 0) left atrial (LA) cell in control. Panel B: Quantification of T-tubules in LVM (n = 23) and LA myocytes (n = 19) using the ratio of the T-tubule region and total cell fluorescence. LA myocytes have less uniform T-tubule structures compared to LVM (***p<0.0001, n = 19).
(TIF)

S1 Text (DOCX)
