# Supplementary figures and images for: Free Fatty Acid Effects on the Atrial Myocardium: Membrane Ionic Currents Are Remodeled by the Disruption of T-Tubular Architecture
Source: PLoS One. 2015 Aug 14;10(8):e0133052. doi: 10.1371/journal.pone.0133052 (PMC4537212; doi:10.1371/journal.pone.0133052)

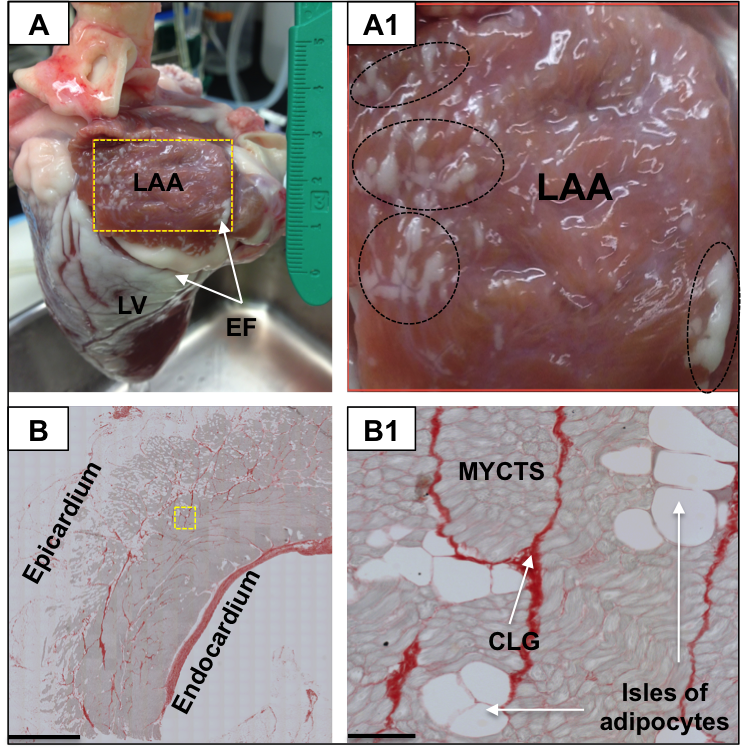

Supplement: S1 Fig — Panel A: Normal adult sheep heart (Langendorff retrograde perfusion), showing epicardial (atrial and ventricular) fat deposits. (LAA; left atrial appendage LV; left ventricle EF; epicardial fat on atrial and ventricular surfaces). Green scale (cm) is shown close to the posterior wall, adjacent to the openings of the pulmonary veins. Yellow box is 2 x 3 cm. Panel A1: (inset from Panel A) oval and rounded rectangles represent regions of dense atrial epicardial fat. Panel B: LAA tissue section: Note significant epicardial fat layer with extensive adipocyte infiltration of the left atrium. Scale bar: 1mm. Panel B1: Tissue section from yellow inset in panel B showing myocytes (MYCTS), isles of adipocytes, and collagen (CLG) stained with Picoserius. Scale bar: 50 μm. (TIFF) [file pone.0133052.s001.tiff]

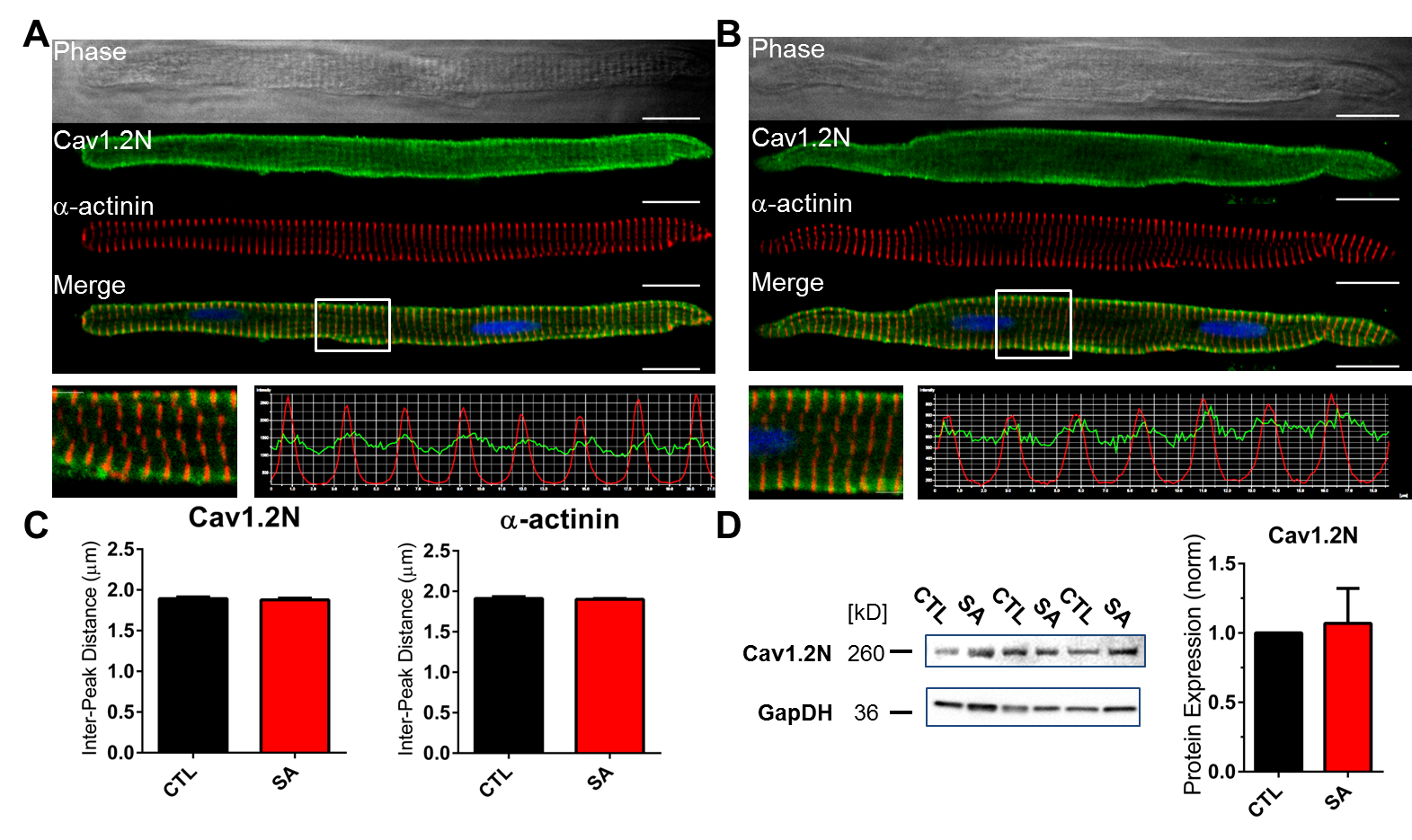

Supplement: S3 Fig — Panel A/B: CTL and SA immunofluorescence images of transmitted light, Cav1.2 nitrosylation (Cav1.2N), α-actinin and a merged image (n = 23, 20) Scale bars: 20 μm. Panel C: Quantification of the intensity profiles shows SA did not alter the mean distance between intensity peaks for Cav1.2N (left) or α-actinin (right; n = 12, 12). Mean distance between Cav1.2N and α-actinin peaks were similar in both groups and unchanged from CTL to SA. Panel D: (left) Western blotting for Cav1.2N with GAPDH as a control. Pane D: (right) normalized densitometry plot of Cav1.2N protein levels in CTL and SA treated cell lysates (N = 3). (TIF) [file pone.0133052.s003.tif]

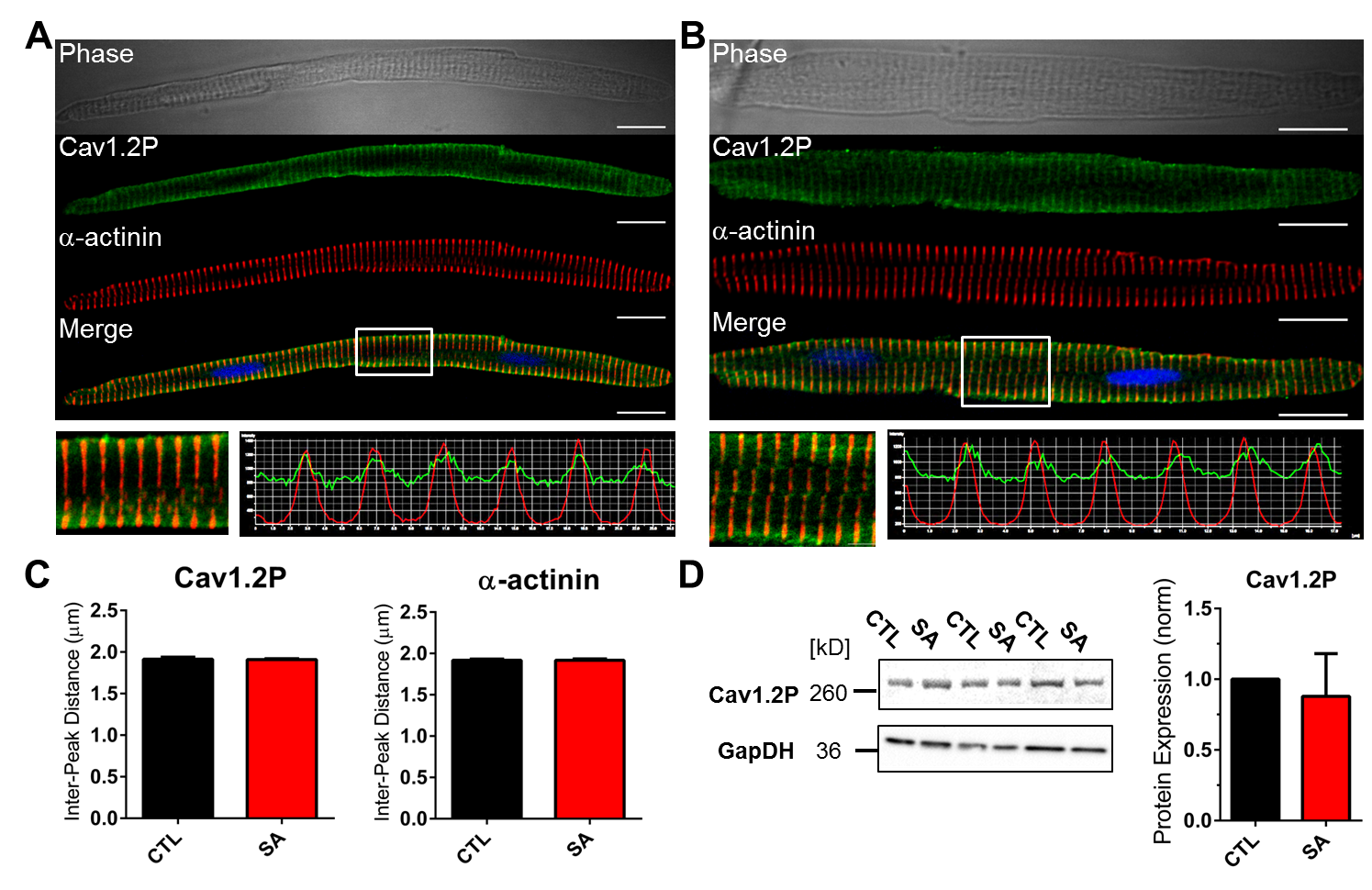

Supplement: S4 Fig — Panel A/B: Immunofluorescence images of transmitted light, Cav1.2 phosphorylation (Cav1.2P), α-actinin and a merged image under control (CTL) and stearic acid (SA) treatment (n = 24, 22). Scale bars: 20 μm. Panel C: Quantification of the intensity profiles shows SA did not alter the mean distance between intensity peaks for Cav1.2P (left) or α-actinin (right; n = 12, 12). Mean distance between Cav1.2P and α-actinin peaks were similar in both groups and unchanged from CTL to SA. Panel D: (left) Western blot for Cav1.2P with GAPDH as a control. Pane D: (right) normalized densitometry plot of Cav1.2P protein levels in CTL and SA treated cell lysates (N = 3). (TIF) [file pone.0133052.s004.tif]

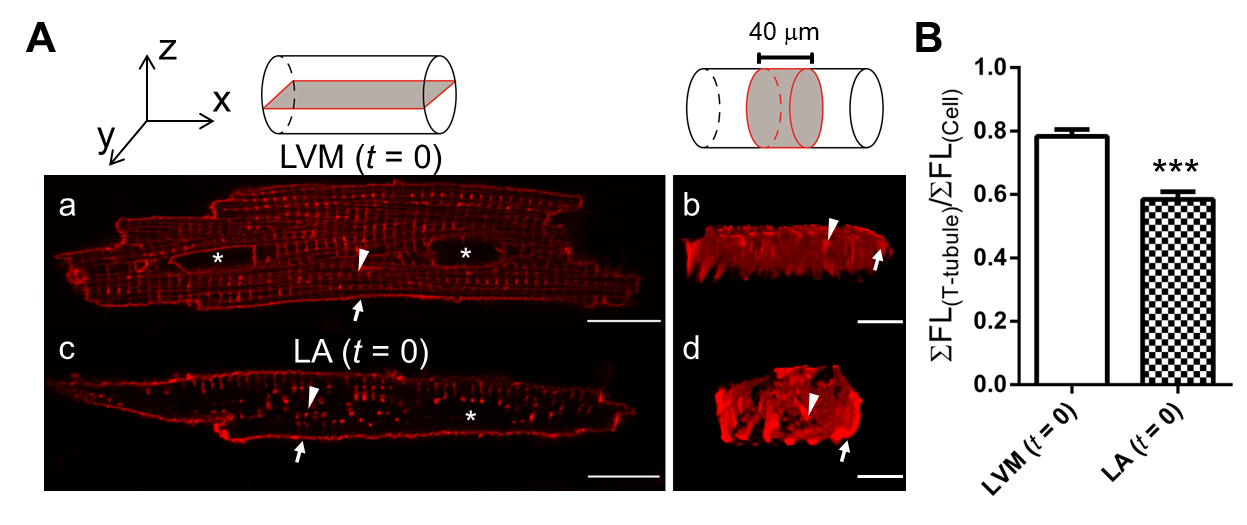

Supplement: S5 Fig — Panel A (top): Coordinate axis for reference and schematic diagrams illustrating the fields of view. Panel A: subpanels (a) and (c) are XY planar views and (b) and (d) are 40 μm ZY cross-sectional views of the same cell. (a) and (b) Di-8-anneps stain of T-tubules in a freshly dissociated (t = 0) left ventricular myocyte (LVM) which contains an extensive T-tubule network. For comparisons, (c) and (d) show a freshly dissociated (t = 0) left atrial (LA) cell in control. Panel B: Quantification of T-tubules in LVM (n = 23) and LA myocytes (n = 19) using the ratio of the T-tubule region and total cell fluorescence. LA myocytes have less uniform T-tubule structures compared to LVM (***p<0.0001, n = 19). (TIF) [file pone.0133052.s005.tif]
