# Supplementary material for: Free Fatty Acid Effects on the Atrial Myocardium: Membrane Ionic Currents Are Remodeled by the Disruption of T-Tubular Architecture
Source: PLoS One. 2015 Aug 14;10(8):e0133052. doi: 10.1371/journal.pone.0133052 (PMC4537212; doi:10.1371/journal.pone.0133052)
Supplement: S1 Text — (DOCX) [file pone.0133052.s006.docx]

**Free fatty acid effects on the atrial myocardium: Membrane ionic currents are remodeled by the disruption of t-tubular architecture**

Ryan P O’Connell PhD^1,2^, Hassan Musa PhD^2^, Mario San Martin Gomez MD, PhD^2^, Uma Mahesh Avula MD^2^, Todd J Herron PhD^1,2^, Jerome Kalifa MD, PhD^2^ and Justus MB Anumonwo PhD^1,2^

Department of Molecular and Integrative Physiology^1^, Center for Arrhythmia Research, Department of Internal Medicine^2^, University of Michigan Medical School, Ann Arbor, MI 48109

**ONLINE SUPPLEMENT**

**Supplemental Results**

*Acute exposure of fatty acids does not affect action potential morphology*

Acute perfusion of PA on LA myocytes did not alter action potential morphology as shown in Supplemental Figure 5A. Quantification of the data revealed no differences at all values measured shown in Supplemental Figure 5B (APD30: CTL: 44.32 ± 6.909 vs. PA: 42.34 ± 8.847; APD50: CTL: 76.33 ± 11.54 vs. PA: 73.83 ± 10.20; APD80: CTL: 144.5 ± 15.61 vs. PA: 138.3 ± 13.89 msec (CTL: N=3, n=13; PA: N=3, n=15). We also acutely perfused SA and found it did not alter the electrophysiology of LA cells, as shown in Supplemental Figure 5C. Quantification of the data yielded no differences between CTL and acute SA treatment shown in Supplemental Figure 5D (APD30: CTL: 38.31 ± 6.907 vs. SA: 39.65 ± 5.099; APD50: CTL: 71.23 ± 11.71 vs. SA: 68.93 ± 7.627; APD80: CTL: 140.2 ± 15.77 vs. SA: 132.4 ± 14.05 msec (CTL: N=3, n=13; SA: N=3, n=13). To determine whether SA had short-term effects on LA myocyte action potentials we incubated SA on cells for 4-6 hours. Supplemental Figure 5E are representative action potential recordings from short-term cultured cells. Quantification of the data showed no differences between CTL and short-term SA incubated cells shown in Supplemental Figure 5F (APD30: CTL: 40.71 ± 7.236 vs. SA: 43.24 ± 6.892; APD50: CTL: 73.61 ± 10.49 vs. SA: 74.00 ± 9.692; APD80: CTL: 145.8 ± 18.94 vs. SA: 151.9 ± 13.79 msec (CTL: N=2, n=7; SA: N=2, n=9).
